# Potential causal associations of PM2.5 and osteoporosis: a two-sample mendelian randomization study

**DOI:** 10.3389/fgene.2024.1263916

**Published:** 2024-02-22

**Authors:** Yi Zhang, Jinsheng Yu, Hang Pei, Xinzheng Zhao, Chao Wang, Guanyin Wang, Zan Shen, Jiang Hua, Bangjian He

**Affiliations:** ^1^ Zhejiang Chinese Medical University, Hangzhou, China; ^2^ Anji County Hospital of Traditional Chinese Medicine, Anji, China; ^3^ First Affiliated Hospital, Zhejiang Chinese Medical University, Hangzhou, China

**Keywords:** PM2.5, air pollution, osteoporosis, mendelian randomization, bone mineral density

## Abstract

**Background:** Observational studies suggest a potential association between atmospheric particulate matter 2.5 (PM2.5) and osteoporosis, but a causal association is unclear due to the presence of confounding factors.

**Methods:** We utilized bone mineral density indices at four specific sites to represent osteoporosis: femoral neck (FN-BMD), lumbar spine (LS-BMD), forearm (FA-BMD), and heel (HE-BMD). The PM2.5 data was obtained from the UK Biobank database, while the datasets for FN-BMD, LS-BMD, and FA-BMD were obtained from the GEFOS database, and the dataset for HE-BMD was obtained from the EBI database. A two-sample Mendelian randomization analysis was conducted using mainly the inverse variance weighted method, horizontal pleiotropy and heterogeneity were also assessed.

**Results:** The results indicated that PM2.5 was not correlated with a decrease in FN-BMD (β: −0.305, 95%CI: −0.762, 0.153), LS-BMD (β: 0.134, 95%CI: −0.396, 0.666), FA-BMD (β: -0.056, 95%CI: −1.172,1.060), and HE-BMD (β: −0.084, 95%CI: −0.261,0.093). Additionally, acceptable levels of horizontal pleiotropy and heterogeneity were observed.

**Conclusion:** In contrast to most observational studies, our research did not discover a potential causal relationship between PM2.5 and the development of osteoporosis.

## Introduction

Osteoporosis stands as a prevalent orthopedic condition, characterized by a decrease in the mineral and bone tissue content of the bone and an increased risk of fracture, it predominantly affects the elderly population, particularly postmenopausal women (Camacho et al., 2020). Fragility fracture is a primary complication of osteoporosis, with a mortality rate as high as 30% within 1 year following the occurrence of the fracture (Sabri et al., 2023). In China alone, with the progression of societal aging, the number of individuals afflicted with osteoporosis has exceeded 90 million, it is estimated that by the year 2035, the healthcare expenditure for the treatment of fractures caused by osteoporosis in China will reach a staggering 199.2 million dollar (Si et al., 2015; Wang et al., 2021).

PM2.5 refers to particulate matter in the atmosphere with a diameter equal to or smaller than 2.5 μm, generated from both human activities and natural environmental processes (Fermo et al., 2019). Due to its extremely small particle size, PM2.5 can be absorbed by the alveoli of the human lungs and enter the bloodstream, posing a significant health risk to people (Huff et al., 2019). Research indicates that PM2.5 can stimulate the release of inflammatory factors and induce cellular carcinogenesis, long-term exposure to PM2.5 has been closely associated with increased incidence of cardiovascular, respiratory, and neurological diseases (Pang et al., 2021).

Common risk factors for osteoporosis include advanced age, BMI, alcohol consumption, high-dose hormones and so on (Kanis et al., 2019; Arceo-Mendoza and Camacho, 2021). In recent years, the impact of living environment on osteoporosis has received extensive attention. As one of the prominent air pollutants, the causal relationship between PM2.5 and osteoporosis has been a subject of controversy, different observational studies and prospective cohort studies have produced conflicting results regarding this association (Chen et al., 2015; Lin et al., 2020; Qiao et al., 2020). This discrepancy may be influenced by confounding factors such as regional variations, lifestyle habits, and ethnic differences, which are difficult to control in traditional research studies (Wilson, 2019; Jones et al., 2020).

Mendelian randomization (MR) is a research method that utilizes genetic variation as a tool to assess the relationship between exposure and outcome. It provides an effective means to mitigate the impact of confounding factors in observational and randomized controlled studies (Bowden and Holmes, 2019). Based on the MR method, multiple SNPs representing PM2.5 exposure (rs1537371; rs77205736, etc.) have been confirmed to be associated with diseases such as hypothyroidism, heart palpitations, and stroke (Zhang et al., 2022). Currently, there is a lack of relevant studies that have conducted an association analysis between PM2.5 and osteoporosis. Therefore, in this study, we aim to investigate whether PM2.5 exposure is a risk factor for osteoporosis by employing a two-sample Mendelian randomization research method, examining the relationship between PM2.5 exposure and bone mineral density (BMD) at multiple sites femoral neck (FN-BMD), lumbar spine (LS-BMD), forearm (FA-BMD), and heel (HE-BMD)).

## Methods

### Research design

**FIGURE 1:**
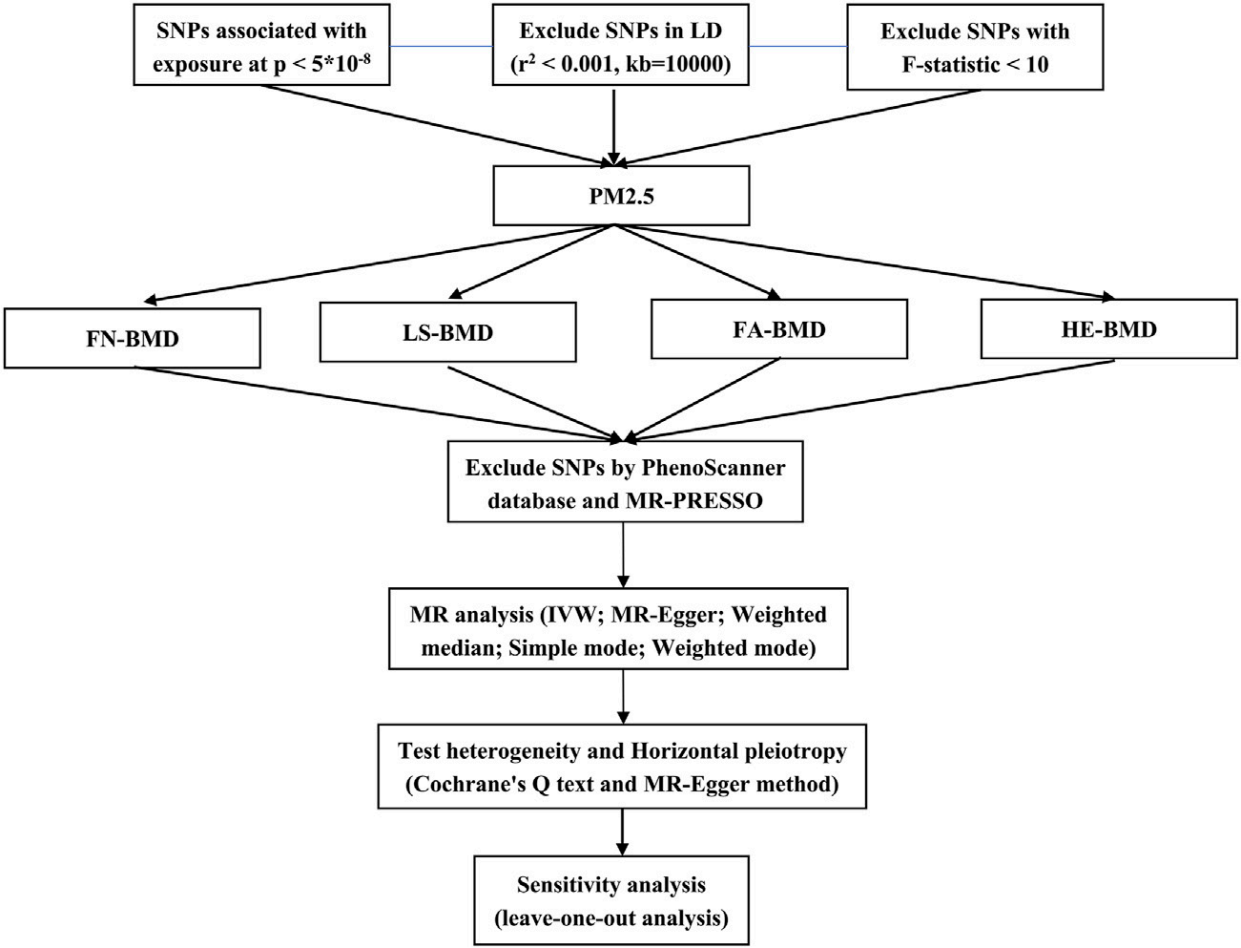
Mendelian randomization flowchart.

This study employed two-sample Mendelian randomization analysis using the pooled genome-wide association studies (GWAS) data set to evaluate the potential causal relationship between PM2.5 and osteoporosis. Additionally, sensitivity analysis was conducted to assess the robustness and reliability of the findings. The specific process was shown in Figure 1.

### Data sources

The GWAS data on PM2.5 exposure used in this study were obtained from the United Kingdom Biobank database (www.ukbiobank.ac.uk), a comprehensive dataset published in 2010, which included a total of 423,796 participants from the European region. The data used in this study is derived from the ESCAPE project (European Study of Cohorts for Air Pollution Effects) (Eeftens et al., 2012).

In clinical practice, osteoporosis is primarily diagnosed using dual-energy X-ray absorptiometry. (DXA) (Camacho et al., 2020). The BMD data for the femoral neck, lumbar spine, and forearm were obtained from the data published by the Genetic Factors for Osteoporosis Consortium website (GEFOS) in 2015, which included a total of 53,236 participants of European ancestry (Zheng et al., 2015). The data on heel bone density was obtained from a large-scale GWAS study that included 583,314 participants of European ancestry (Loh et al., 2018). The above data can be downloaded from IEU OpenGWAS project. For specific data, see Supplementary Material S1.

### Instrumental variables

Mendelian randomization analysis relies on three core principles that must be met: 1) The instrumental variable must have a strong correlation with the exposure factor; 2) The instrumental variable should not be associated with confounding factors; 3) The instrumental variable’s value can only affect the outcome variable through the exposure factor (Bowden and Holmes, 2019).

To satisfy the principles, we conducted SNPs that showed significant correlation with PM2.5 at the genome-wide level (*p* < 5*10^–8^, *r* ^2^ < 0.001, and kb = 10,000) (Wu et al., 2020). Then, to mitigate the risk of weak instrumental variables, we employed F-statistic (F = *R* ^2^ (n − k − 1)/k (1 −*R* ^2^)) to assess their presence (F < 10 indicates the presence of weak instrumental variables (IVs)) (Burgess et al., 2011). After excluding SNPs in linkage disequilibrium, we harmonized the data for the exposure and outcome SNPs. We then performed a manual search using the PhenoScanner database (www.phenoscanner.medschl.cam.ac.uk/) to determine whether the included SNPs were associated with confounding factors (*p* < 1*10^–5^).

### Analysis strategy

After screening the relevant SNPs, we used the MR-PRESSO method to test the outliers in all the results. If *p* < 0.05, the SNP was considered as an outlier SNP and deleted to reduce the level of pleiotropy caused by it. Then, we employed the Inverse Variance Weighted method (IVW) to combine the effect sizes of SNPs (using a random-effects model when the exposure was assessed by at least 3 SNPs; otherwise, a fixed-effects model was used). Additionally, we utilized the Weighted Median and MR-Egger methods as supplementary approaches to validate the results (Hemani et al., 2018). Finally, we used Cochrane’s Q text and MR-Egger method (by intercept tests) to test heterogeneity and horizontal pleiotropy (Burgess and Thompson, 2017). In addition, the leave-one-out analysis was carried out by sequentially eliminating each instrumental variable to assess whether any single SNP disproportionately influenced the results.

Since the outcome was a continuous variable, this study reports the β values and their 95% confidence intervals. All statistical analyses were conducted using R software (version 4.0.3) with the assistance of the “TwoSampleMR” and “MR-PRESSO” packages. In addition, to exclude the interference of the false positive rate, we use the Bonferroni method to re-correct the threshold (*p* < 0.0125 is statistically significant).

## Results

After removing SNPs in linkage disequilibrium, we identified 8 SNPs that were significantly associated with PM2.5 exposure in the GWAS studies. We manually searched for confounding SNPs and excluded two confounding SNPs (rs114708313 and rs77205736) that was related to rheumatoid arthritis and BMI. After matching with the bone density data of the four sites, we obtained a final set of 6 IVs (specific data in Table 1). After calculating, all IVs yielded F-values greater than 10, indicating the absence of weak IVs.

**TABLE 1:** include specific data for SNPs.

Exposure	Outcomes	Pleiotropy test			Heterogeneity test					
		MR-Egger			MR-Egger			IVW		
		Intercept	SE	*p*-value	Q	Q_df	Q_pval	Q	Q_df	Q_pval
PM2.5	FA-BMD	−0.018	0.019	0.407	4.134	3	0.247	5.408	4	0.248
	FN_BMD	−0.009	0.007	0.302	2.257	3	0.521	3.806	4	0.432
	LS_BMD	−0.002	0.009	0.866	0.828	3	0.843	0.862	4	0.930
	HE_BMD	0.002	0.004	0.533	11.028	4	0.026	12.307	5	0.031

**FIGURE 2:**
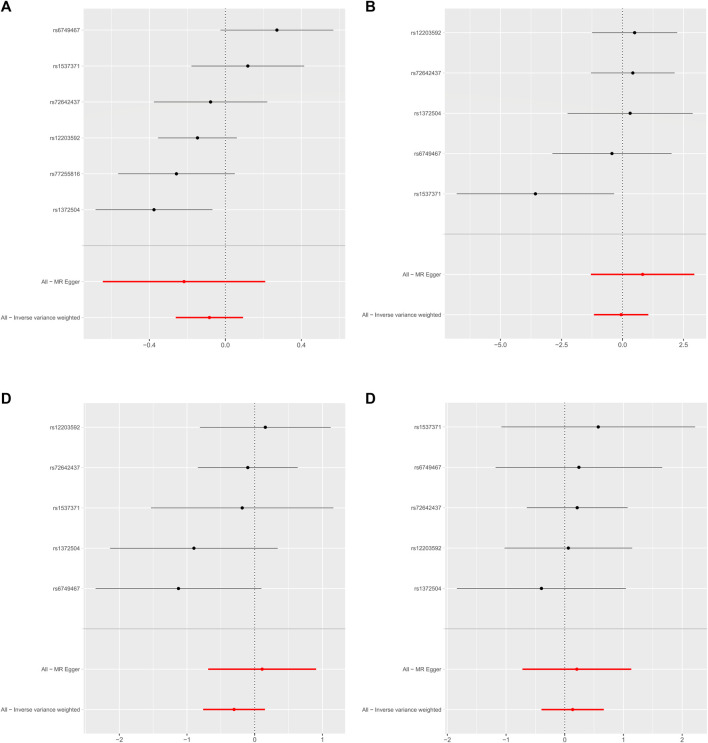
Forest plot of mendelian randomization Analysis. **(A)** HE-BMD, **(B)** FA-BMD, **(C)** FN-BMD, **(D)** LS-BMD.

**FIGURE 3:**
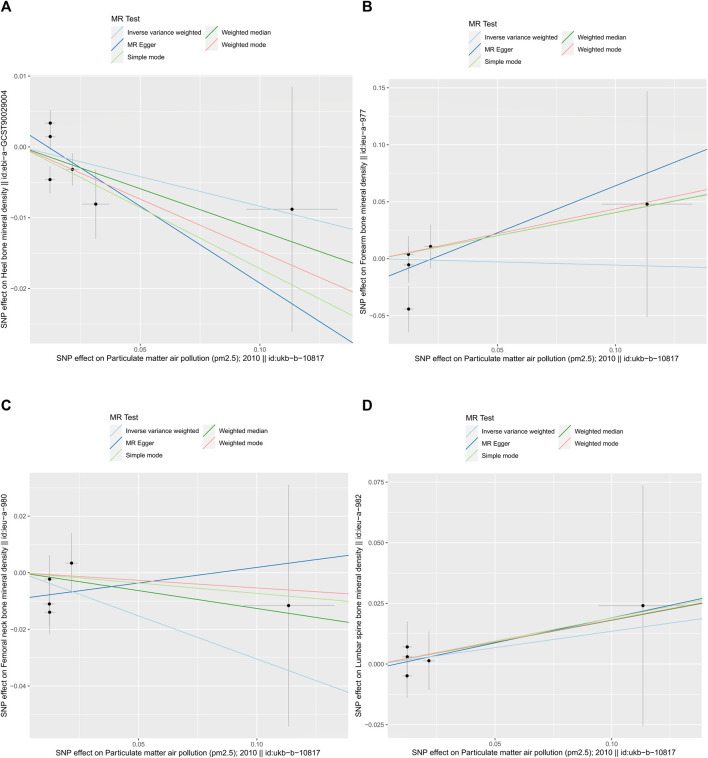
summary plot of MR-Egger method (by intercept tests) data. **(A)** HE-BMD, **(B)** FA-BMD, **(C)** FN-BMD, **(D)** LS-BMD.

**FIGURE 4:**
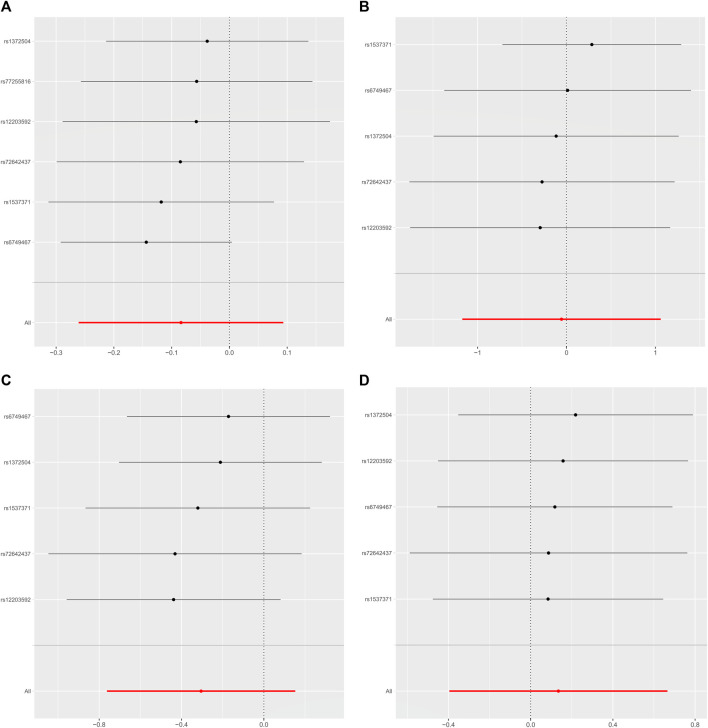
Leave-one-out data plot of the final included SNPs. **(A)** HE-BMD, **(B)** FA-BMD, **(C)** FN-BMD, **(D)** LS-BMD.

As shown in Table 2, the IVW suggests that the exposure to PM2.5 environment has no correlation with the changes in FN-BMD (β: −0.305, 95%CI: −0.762, 0.153; P: 0.191), LS-BMD (β: 0.134, 95%CI: −0.396, 0.666; P: 0.618), FA-BMD (β: −0.056, 95%CI: −1.172,1.060; P: 0.922), and HE-BMD (β: −0.084, 95%CI: −0.261,0.093; P: 0.353). The weighted median method and MR-Egger also showed the same results (shown in Figure 2). Both the Cochrane’s Q test and the MR-Egger method indicate the absence of horizontal pleiotropy and heterogeneity were observed (specific data in Table 3 and Figure 3). Furthermore, during the sensitivity analysis of the Mendelian randomization (MR) results using the leave-one-out method, we did not observe any causal shift (shown in Figure 4).

**TABLE 2:** specific data for Mendelian randomization Analysis.

SNPs	β	SE	*p*-Value	β	SE	*p*-Value	β	SE	*p*-Value	β	SE	*p*-Value	Gene	Function
	FA-BMD			LS-BMD			FN-BMD			HE-BMD				
rs12203592	0.496	0.890	0.577	0.062	0.555	0.911	0.157	0.493	0.750	−0.147	0.106	0.165	IRF4	intron
rs1372504	0.312	1.307	0.811	−0.397	0.734	0.589	−0.898	0.632	0.155	−0.376	0.157	0.017	RP11-6N13.1	intron
rs1537371	−3.568	1.646	0.030	0.571	0.842	0.498	−0.184	0.687	0.789	0.118	0.152	0.435	CDKN2B-AS1	intron
rs6749467	−0.432	1.246	0.729	0.241	0.724	0.739	−1.126	0.625	0.072	0.272	0.152	0.073	LINC01865	downstream
rs72642437	0.422	0.874	0.629	0.212	0.438	0.628	−0.102	0.376	0.786	−0.078	0.152	0.610	ZBTB7C	intron
rs77255816	—	—	—	—	—	—	—	—	—	−0.257	0.157	0.111	CDKAL1	intron

**TABLE 3:** the data of Pleiotropy test and Heterogeneity test.

Exposure	Outcomes	N (SNPs)	Methods	Beta	SE	OR (95%CI)	*p*-value
PM2.5	FA-BMD	5	MR Egger	0.825	1.081	0.274, 18.990	0.501
		5	Weighted median	0.405	0.594	0.469, 4.801	0.495
		5	Inverse variance weighted	−0.056	0.569	0.310, 2.887	0.922
		5	Simple mode	0.407	0.663	0.410, 5.511	0.572
		5	Weighted mode	0.437	0.635	0.446, 5.371	0.529
	FN-BMD	5	MR Egger	0.110	0.407	0.503, 2.479	0.804
		5	Weighted median	−0.126	0.295	0.494, 1.573	0.669
		5	Inverse variance weighted	−0.305	0.233	0.467, 1.165	0.191
		5	Simple mode	−0.073	0.416	0.411, 2.102	0.870
		5	Weighted mode	−0.054	0.351	0.477, 1.885	0.886
	LS-BMD	5	MR Egger	0.206	0.473	0.486, 3.105	0.692
		5	Weighted median	0.180	0.318	0.643, 2.232	0.570
		5	Inverse variance weighted	0.135	0.271	0.673, 1.946	0.618
		5	Simple mode	0.190	0.417	0.534, 2.735	0.672
		5	Weighted mode	0.182	0.376	0.575, 2.507	0.652
	HE-BMD	6	MR Egger	−0.217	0.218	0.525, 1.234	0.375
		6	Weighted median	−0.118	0.081	0.758, 1.042	0.145
		6	Inverse variance weighted	−0.084	0.090	0.770, 1.098	0.354
		6	Simple mode	−0.172	0.138	0.643, 1.104	0.268
		6	Weighted mode	−0.148	0.110	0.696, 1.070	0.236

## Discussion

To our knowledge, this study represents the inaugural exploration of the causal relationship between PM2.5 exposure and osteoporosis. We explored the potential impact of PM2.5 on HE-BMD, FA-BMD, FN-BMD, and LS-BMD. The results showed that after correction for multiple comparisons, no causal relationship between PM2.5 and osteoporosis was found.

So far, there is no conclusion on the relationship between PM2.5 and osteoporosis. An Italian cohort study of 59,950 female participants found that exposure to particulate matter caused a decrease in bone density, particularly in the femoral neck (Adami et al., 2022). Simultaneously, a cross-sectional study focused on males similarly observed a declining trend in bone density after prolonged exposure to PM2.5-10 environments (Alvaer et al., 2007). Intriguingly, two extensive database studies conducted in the United Kingdom and China similarly identified PM2.5 exposure as a risk factor for osteoporotic fractures (Chiu et al., 2021; Shi et al., 2022). Contrary to these, Heo et al. and Alver et al. found no changes in bone density or osteoporotic fracture risk associated with short-term or long-term exposure to PM2.5 (Alver et al., 2010; Heo et al., 2022). Similarly, our study did not identify a potential causal relationship between them. In observational studies, whether it is multi-factor regression or other large-sample prediction models, it is impossible to eliminate the influence of confounding factors, such as alcohol abuse, smoking, high-fat diet, etc (Aibar-Almazan et al., 2022). Concurrently, drug utilization is another crucial influencing factor, the usage of hormones as common respiratory medications significantly rises in regions with severe air pollution (Liu et al., 2022). However, our investigation did not identify SNPs associated with confounders in the included IVs, suggesting that our study was not affected by the confounders mentioned above. It is noteworthy that due to the diversity of sources, there are considerable variations in PM2.5 components across different regions, making it challenging to avoid regional disparities in all studies (Albinet et al., 2007; Shah et al., 2013).

Due to its small particle size, PM2.5 can penetrate the alveoli and enter the bloodstream, leading to potential harm to various organs in the body (Thiankhaw et al., 2022). Research has indicated that PM2.5 can trigger oxidative stress and inflammation in the compromised airways through pathways involving protein kinases and Toll-like receptors (Guo et al., 2017). This activation can result in the upregulation of inflammatory markers such as TNF-α, IL-17, and RANKL, which in turn may lead to enhanced bone resorption (Shen et al., 2005; Saha et al., 2016). Postmenopausal women, due to the decline in estrogen levels, experience a diminished capacity to inhibit osteoclasts, making them a high-risk population for osteoporosis (Singh et al., 2022). Furthermore, several studies have observed that exposure to PM2.5 can induce apoptosis in follicle cells, consequently leading to a decline in ovarian reserve function, this decline in ovarian function may contribute to an increased incidence of osteoporosis in women (Luderer et al., 2022). These findings further underscore the potential impact of PM2.5 on women’s skeletal health. Vitamin D (VD), an essential nutrient for calcium circulation in the human body, is synthesized predominantly (95%) through the skin (Wintermeyer et al., 2016). The results of our study indicate that rs12203592 may be a crucial locus linking PM2.5 exposure to osteoporosis. Previous research suggests that rs12203592, located on chromosome 6 near the IRF4 gene, plays a regulatory role in skin pigmentation and modulation of the VD receptor, affecting the synthesis of VD precursors (Ramagopalan et al., 2010). Additionally, some researchers propose an association between PM2.5 exposure and VD deficiency, potentially linked to PM2.5-induced renal damage and the presence of polycyclic aromatic hydrocarbons in PM2.5 accelerating VD degradation (Calderon-Garciduenas et al., 2015; Xu et al., 2023). While numerous experimental studies suggest a potential association between PM2.5 and osteoporosis, our MR analysis within the studied population did not reveal such a link, warranting further validation.

Our study has several strengths. First, the MR method effectively circumvents confounding factors and reverse causation prevalent in traditional observational studies. Furthermore, we applied more stringent confounding factor exclusion criteria (*p* < 1*10^–5^). Second, the instrumental variables (IVs) for PM2.5 exposure and osteoporosis were concurrently derived from existing large-scale GWAS, allowing for a more precise assessment of effect sizes compared to individual-level data or studies with limited sample sizes. However, it is important to acknowledge some potential limitations. The dataset used in our study has limitations in population stratification: there was no stratification based on age and gender, restricting our ability to analyze and identify populations at risk of osteoporosis under PM2.5 exposure. Future research should consider datasets with finer population segmentation for more accurate conclusions through stratified analysis. Besides, due to limitations in GWAS data, our study only supports the causal relationship between PM2.5 exposure and osteoporosis in the European population. Subsequent investigations should utilize larger sample data from different populations for in-depth analysis.

## Conclusion

In summary, this study verified that there is no necessary relationship between PM2.5 exposure and the risk of osteoporosis through Mendelian randomization analysis. In addition, due to differences in the environment and population genes in different regions, more up-to-date data are needed for verification.

## Data Availability

The original contributions presented in the study are included in the article/Supplementary Material, further inquiries can be directed to the corresponding author.
