# Supplementary material for: Potential causal associations of PM2.5 and osteoporosis: a two-sample mendelian randomization study
Source: Front Genet. 2024 Feb 22;15:1263916. doi: 10.3389/fgene.2024.1263916 (PMC10921569; doi:10.3389/fgene.2024.1263916)
Supplement: Supplementary file 1 [file DataSheet1.docx]

|  | ID | Sample size | Number of SNPs | Consortium | Platform | chip |
| --- | --- | --- | --- | --- | --- | --- |
| PM2.5 | ukb-b-10817 | 423,796 | 9,851,867 | MRC-IEU | NA | NA |
| FN-BMD | ieu-a-980 | 32,735 | 10,586,900 | GEFOS | Illumina HiSeq | NA |
| LS-BMD | ieu-a-982 | 28,498 | 10,582,867 | GEFOS | Illumina HiSeq | NA |
| FA-BMD | ieu-a-977 | 8,143 | 9,955,366 | GEFOS | Illumina HiSeq | NA |
| HE-BMD | ebi-a-GCST90029004 | 583,314 | 11,972,309 | NA | NA | NA |
